# Analgesic effect of pulsed electromagnetic fields for mammaplasty: A meta-analysis of randomized controlled studies

**DOI:** 10.1097/MD.0000000000021449

**Published:** 2020-08-28

**Authors:** Li Zhang, Wei Ding, Yu Ji

**Affiliations:** a Department of Plastic Surgery, The First people's Hospital of Xiaoshan; b Department of Plastic Surgery, Zhejiang Provincial People's Hospital, People's Hospital of Hangzhou Medical College, Hangzhou; Zhejiang Province, P.R. China.

**Keywords:** mammaplasty, pain control, pulsed electromagnetic fields, randomized controlled trials

## Abstract

**Background:**

Pulsed electromagnetic fields shows some potential in alleviating pain after mammaplasty. This systematic review and meta-analysis is conducted to investigate the analgesic efficacy of pulsed electromagnetic fields for pain control after mammaplasty.

**Methods:**

The databases including PubMed, EMbase, Web of science, EBSCO, and Cochrane library databases are systematically searched for collecting the randomized controlled trials regarding the impact of pulsed electromagnetic fields on pain intensity after mammaplasty.

**Results:**

This meta-analysis has included 4 randomized controlled trials. Compared with control group after mammaplasty, pulsed electromagnetic fields results in remarkably reduced pain scores on 1 day (MD = −1.34; 95% confidence interval [CI] = −2.23 to −0.45; *P* = .003) and 3 days (MD = −1.86; 95% CI = −3.23 to −0.49; *P* = .008), as well as analgesic consumption (Std. MD = −5.64; 95% CI = −7.26 to −4.02; *P* < .00001).

**Conclusions:**

Pulsed electromagnetic fields is associated with substantially reduced pain intensity after mammaplasty.

## 1 Introduction

Acute postoperative pain widely occurred in patients with mammaplasty such as breast reconstruction and augmentation.^[1–4]^ This postoperative pain results in poor recovery and the development of chronic postoperative pain.^[5–7]^ Many methods have been developed to reduce postsurgical pain after mammaplasty, but there are lack of consistent benefits such as intravenous analgesics, indwelling pain catheters, and simple irrigation or infiltration of local anesthetics.^[8–11]^

Pulsed electromagnetic fields have been a successfully noninvasive and nonthermal approach to accelerate the repair of delayed and nonunion fractures and chronic wounds, and the reduction of pain and inflammation.^[12–14]^ Postoperative pain control of breast surgery has become a significant concern for surgeons.^[15–18]^ In patients with breast augmentation and reduction, disposable pulsed electromagnetic field devices applied immediately after the surgery, can significantly accelerate pain reduction and reduce postoperative narcotic requirements.^[19–21]^

Recently, several studies regarding the effect of pulsed electromagnetic fields on pain intensity after mammaplasty have been published, and the results have not been well established.^[21–23]^ Considering these inconsistent effects, we therefore conduct a systematic review and meta-analysis of randomized controlled trials (RCTs) to evaluate the efficacy of pulsed electromagnetic fields on pain control after mammaplasty.

## 2 Materials and methods

Preferred Reporting Items for Systematic Reviews and Meta-analysis statement^[24]^ and the Cochrane Handbook for Systematic Reviews of Interventions^[25]^ are used to guide the performance of this systematic review and meta-analysis. Two investigators have independently searched articles, extracted data, and assessed the quality of included studies, so ethical approval was not necessary.

### 2.1 Literature search and selection criteria

Several databases including PubMed, EMbase, Web of science, EBSCO, and the Cochrane library are systematically searched using the keywords: mammaplasty or breast augmentation or breast reconstruction or breast reduction, and electromagnetic fields. The time in publishing the studies is from inception to February, 2020.

The inclusion criteria are as follows:

(1) study design is RCT,
(2) study population are patients undergoing mammaplasty,
(3) intervention treatments are pulsed electromagnetic fields versus placebo.

### 2.2 Data extraction and outcome measures

Some information is collected for summarizing the baseline characteristics of patients in the included RCTs, and they include first author, publication year, sample size, body mass index, baseline pain scores and detail methods of 2 groups.

The primary outcomes are pain scores on 1 day and 3 days. Secondary outcome is analgesic consumption.

### 2.3 Quality assessment in individual studies

The methodological quality of included RCTs is evaluated using the Jadad Scale, which is composed of 3 evaluation elements including randomization (0–2 points), blinding (0–2 points), dropouts and withdrawals (0–1 points).^[26]^ One point would be allocated to each element based on the description, randomization and/or blinding of the included RCTs. The score of Jadad Scale has a range from 0 to 5 points, and 1 study with Jadad score ≥3 is thought to have the high quality.^[27]^

### 2.4 Statistical analysis

Review Manager Version 5.3 (The Cochrane Collaboration, Software Update, Oxford) is used for the all statistical analyses. We have calculated the mean differences (MDs) or standard mean differences with 95% confidence intervals (CIs) for all continuous outcomes. Heterogeneity is quantified with the *I*^2^ statistic, and an *I*^2^ value greater than 50% represents the significant heterogeneity. The random-effect model with DerSimonian and Laird weights is applied for all the meta-analyses regardless of the heterogeneity. When the significant heterogeneity presents, sensitivity analysis is conducted to detect the influence of a single study on the overall estimate or perform the subgroup analysis. Publication bias is not evaluated because of the limited number (<10). *P* < .05 is thought to be statistically significant.

## 3 Results

### 3.1 Literature search, study characteristics and quality assessment

**Figure 1:**
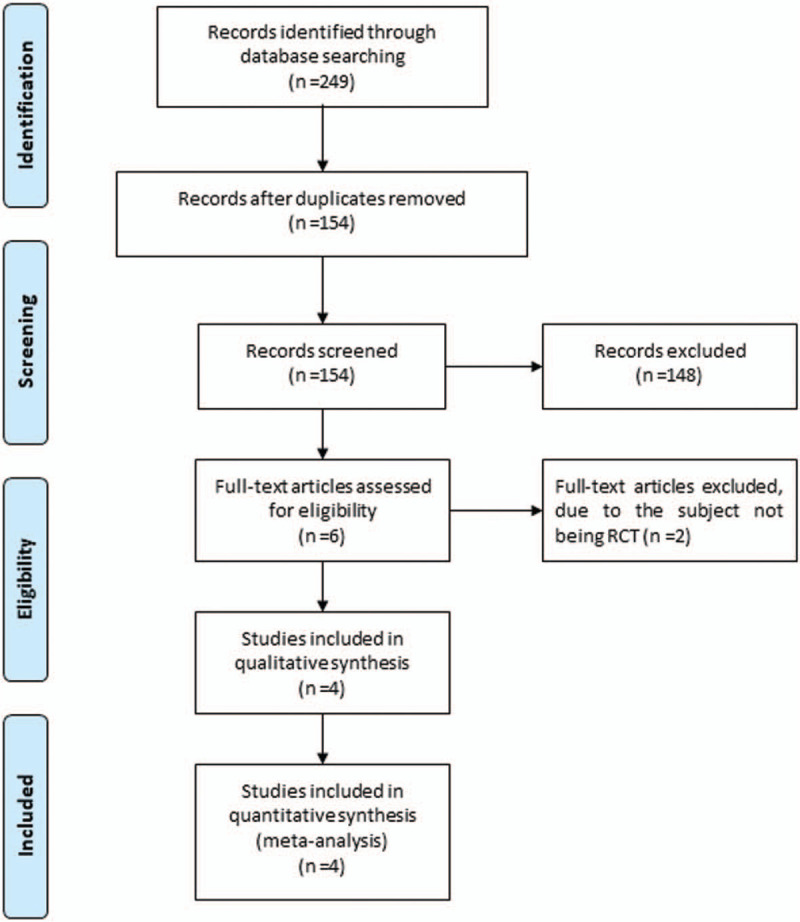
Flow diagram of study searching and selection process.

Figure 1 demonstrates the flow chart for the selection process and detailed identification. 249 publications are searched after the initial search of databases. 95 duplicates and 148 papers after checking the titles/abstracts are excluded. Two studies are removed because of the study design and four RCTs are ultimately included in the meta-analysis.^[19,21–23]^

Table 1 shows the baseline characteristics of 4 eligible RCTs. The 4 studies are published between 2008 and 2016, and total sample size is 164. The surgical procedures include the breast augmentation,^[19,22]^ breast reconstruction,^[23]^ and breast reduction.^[21]^ Among the four RCTs, 2 studies report pain scores on 1 day,^[22,23]^ 2 studies report pain scores on 3 days,^[19,23]^ and four studies report analgesic consumption.^[19,21–23]^ Jadad scores of the four eligible studies vary from 3 to 4, and thus this quality assessment confirms these studies with high-quality.

**Table 1:**
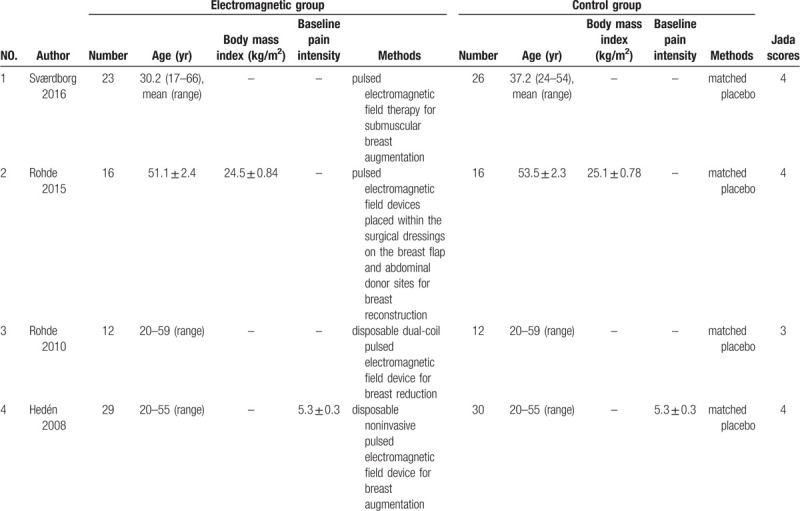
Characteristics of included studies.

### 3.2 Primary outcomes: pain scores on 1 day and 3 days

**Figure 2:**
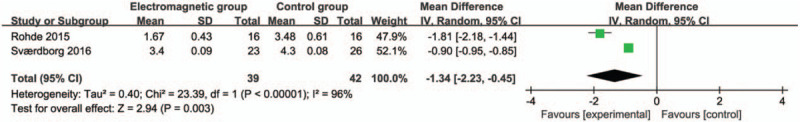
Forest plot for the meta-analysis of pain scores on 1 day.

**Figure 3:**
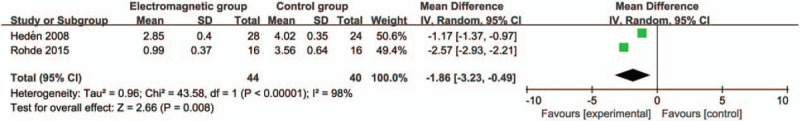
Forest plot for the meta-analysis of pain scores on 3 days.

The random-effect model is used for the analysis of primary outcomes. The results find that compared to control intervention after mammoplasty, pulsed electromagnetic fields is associated with substantially reduced pain scores on 1 day (MD = −1.34; 95% CI = −2.23 to −0.45; *P* = .003) with significant heterogeneity among the studies (*I*^2^ = 96%, heterogeneity *P* < .00001, Fig. 2) and 3 days (MD = −1.86; 95% CI = −3.23 to −0.49; *P* = .008) with significant heterogeneity among the studies (*I*^2^ = 98%, heterogeneity *P* < .00001, Fig. 3).

### 3.3 Sensitivity analysis

**Figure 4:**
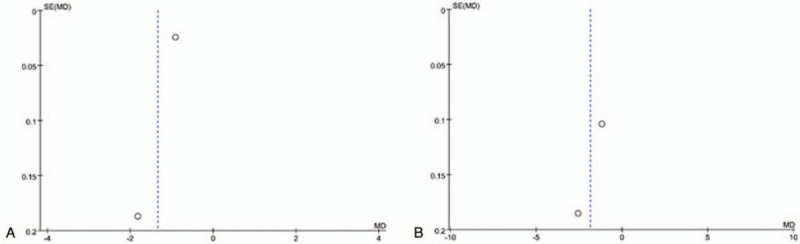
Funnel plot for the outcome of pain scores on 1 day (A) and 3 days (B).

The meta-analysis of pain scores on 1 day and 3 days has significant heterogeneity among the included studies, but there are just 2 studies included. Therefore, we do not perform sensitivity analysis by omitting 1 study in each turn or conduct the subgroup analysis. Figure 4 shows a funnel plot for studies reporting pain scores on 1 day and 3 days. The plot is obviously not symmetrical, which also indicates the significant heterogeneity.

### 3.4 Secondary outcome

**Figure 5:**
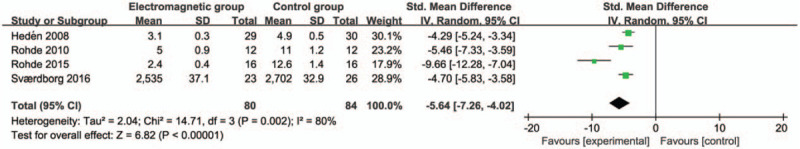
Forest plot for the meta-analysis of analgesic consumption.

In comparison with control intervention after mammaplasty, pulsed electromagnetic fields results in significantly reduced analgesic consumption after the surgery (Std. MD = −5.64; 95% CI = −7.26 to −4.02; *P* < .00001; Fig. 5).

## 4 Discussion

The efficacy of pulsed electromagnetic field therapy on postoperative pain and narcotic use were comparable to those patients with breast augmentation and reduction.^[19–21]^ Pulsed electromagnetic field effect on inflammation and interleukin-1β was similar to breast reconstruction and breast reductions using the same pulsed electromagnetic field signal.^[21,23,28]^ Our meta-analysis suggested that pulsed electromagnetic fields can significantly reduce the pain scores on 1 days and 3 days, as well as the analgesic consumption for patients with breast mammaplasty.

Pulsed electromagnetic fields was documented to significantly decrease the levels of interleukin-1β, and wound exudate volume in the first 24 hours postoperatively. Interleukin-1β was known as a principal inflammatory cytokine involved in pain hypersensitivity in wound exudates.^[21,23]^ The beneficial mechanisms of pulsed electromagnetic fields for pain control remained elucidated, and may be associated with the modulation of calmodulin-dependent nitric oxide/cyclic guanosine monophosphate signaling, a primary anti-inflammatory and repair pathway.^[29–32]^ Nitric oxide/cyclic guanosine monophosphate signaling activated by pulsed electromagnetic fields leads to the decreased release of proinflammatory cytokines (eg, interleukin-1β) and the increased release of anti-inflammatory cytokines (eg, interleukin-6 and interleukin-10) and growth factors (eg, fibroblast growth factor-2) in challenged cells and tissues.^[33–36]^ Pulsed electromagnetic field signal was also reported to enhance microvascular perfusion and neuronal regeneration.^[37,38]^

Previous studies focused on the effect of pulsed electromagnetic field dosing on the competing dynamics of calmodulin-dependent nitric oxide/cyclic guanosine monophosphate signaling and phosphodiesterase inhibition of cyclic guanosine monophosphate on pain outcome in breast reduction patients, and the results revealed that pain outcomes were dependent on the rate of increased nitric oxide in tissue.^[23,39]^ Pulsed electromagnetic field signal consisting of a 2-msec burst of 27.12-MHz radiofrequency sinusoidal waves repeating at 2 bursts/s provided adequate dosing to have a net positive effect on postoperative pain reduction.^[12,23,39]^ Adjunctive pulsed electromagnetic fields can serve as an important tool for the surgeon to accelerate the reduction of postsurgical pain and inflammation, decrease patient morbidity and enhance surgical outcomes.^[23]^

There are still several limitations. First, only 4cd RCTs are included in this meta-analysis, and all of them have a relatively small sample size (n < 100). These may lead to overestimation of the treatment effect in smaller trials. Sec, there is significant heterogeneity among the included studies, which may be caused by different procedures of mammaplasty such as breast augmentation and reduction which produced various pain intensity scales. Finally, different analgesics for pain rescue were used, which may lead to some bias to the pooled effect.

## 5 Conclusion

Pulsed electromagnetic fields can significantly enhance pain relief in patients with mammaplasty.

## Author contributions

XXXX.
